# Tunable two-dimensional polarization grating using a self-organized micropixelated liquid crystal structure†

**DOI:** 10.1039/c8ra08557a

**Published:** 2018-12-12

**Authors:** Reo Amano, Péter Salamon, Shunsuke Yokokawa, Fumiaki Kobayashi, Yuji Sasaki, Shuji Fujii, Ágnes Buka, Fumito Araoka, Hiroshi Orihara

**Affiliations:** Division of Applied Physics, Faculty of Engineering, Hokkaido University North 13 West 8, Kita-ku Sapporo Hokkaido 060-8628 Japan yuji.sasaki@eng.hokudai.ac.jp +81 (0)11 7066642; Institute for Solid State Physics and Optics, Wigner Research Centre for Physics, Hungarian Academy of Sciences H-1525 Budapest P. O. Box 49 Hungary; RIKEN Center for Emergent Matter Science (CEMS) 2-1 Hirosawa Wako, Saitama 351-0198 Japan

## Abstract

Utilization of the self-organizing nature of soft materials is promising for fabricating micro- and nano-structures, which can be applied for optics. Because of the high birefringence, liquid crystals are especially suitable for optoelectronic applications such as beam steering and polarization conversion. On the other hand, most self-organized patterns in liquid crystals are one-dimensional and there are only a few examples of two dimensional systems. Here we study the light diffraction from a micro-pixelated pattern of a nematic liquid crystal which is formed by self-organization of topological defects. We demonstrate that the system works as a tunable two dimensional optical grating, which splits the incident laser beam and changes the polarization property. The intensity can be controlled by electrical voltages, which cause extinction of the zeroth-order beam. The polarization properties depend on the location of spots. The numerical calculation and the theoretical analysis not only support the experimental results but also unveil the uniqueness of the pixelated structure.

## Abstract 2

A micro-pixelated pattern of a nematic liquid crystal formed by self-organization of topological defects is shown to work as a tunable two-dimensional optical grating.

## Graphical abstract

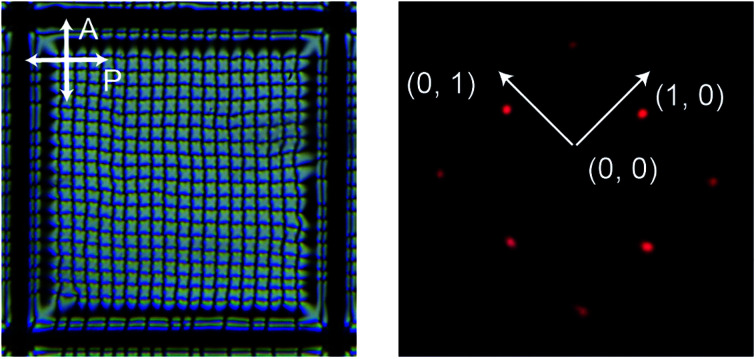

## Introduction

Nematic liquid crystals (NLCs) are anisotropic fluids in which elongated molecules are aligned in a preferred direction called the director, ***n***. Because the optical anisotropy can be controlled by electric fields, NLCs are suitable for optoelectronic applications such as displays. LC gratings are interesting examples, which are targeted to beam steering and optical filters.^1–8^ In particular, LC gratings can show high first-order diffraction efficiency and excellent polarization–separation properties.^1,3,5,9–12^ In order to fabricate gratings using LCs, a periodic modulation of director field in a small area is essentially required. Several methods have been proposed so far to realize high-resolution control of ***n*** for various LCs applications. Common approaches are to use top-down lithographic processes such as pre-patterned electrodes,^1,11,13,14^ scribing the substrate by AFM,^15^ and photo-alignment.^9,16–18^ Another candidate is the bottom-up approach that uses a self-organized periodic pattern.^2,6,19–21^ It is known that LCs generally exhibit rich pattern formation^22–24^ under external stimuli such as the electro-hydrodynamic effect^25,26^ and by the helical structure of the chiral NLCs.^27–29^ However these patterns are mostly limited to one-dimensional striped structure. Two dimensional patterns are relatively rare^23–26^ and moreover it is difficult to obtain a mono-domain structure in a large area. Thus, the diffraction properties of two dimensional self-organized patterns have not been investigated thoroughly. In this regard, recently, by doping an NLC with ionic additives, we have found a two-dimensional micropattern, which is self-organized by applying electric fields.^30^ The pattern consists of a square array of topological defects, which are often called umbilics.^31,32^ The umbilical defect structure is induced when homeotropically aligned NLC molecules with negative dielectric anisotropy are reoriented. Because this system does not require a pre-patterned surface, it is possible to change both the size of the unit cell and the birefringence with external fields. Thus, such a self-organized system is interesting for optical applications. One particular exploitation is the generation of an optical vortex from the defect structure.^33,34^ We have recently reported optical vortex generation using this two-dimensional grid pattern.^35^ There, we have simultaneously observed light diffraction. However, in the earlier paper, the diffraction has not been understood in detail.

In this paper, we present the results of our comprehensive investigation aiming to understand light diffraction from the micro-pixelated LC pattern. We uncover how the diffraction pattern depends on the effective birefringence and on the frequency of the driving AC electrical voltage. The polarization state of the diffracted light is also studied. The experiments show that the intensity of diffraction spots can be controlled by the reorientation of the director field. Particularly, high diffraction-efficiency is realized under a moderate electrical voltage, which extincts the zeroth order spot. We also find that the polarization of diffracted light is converted differently depending on the diffraction order. In addition to experiments, we perform numerical calculations to understand the voltage dependence, which shows a qualitative agreement with the experimental results. The presented experimental and numerical results are understood theoretically, which also reveals the uniqueness of this pixelated structure.

## Experimental

**Fig. 1:**
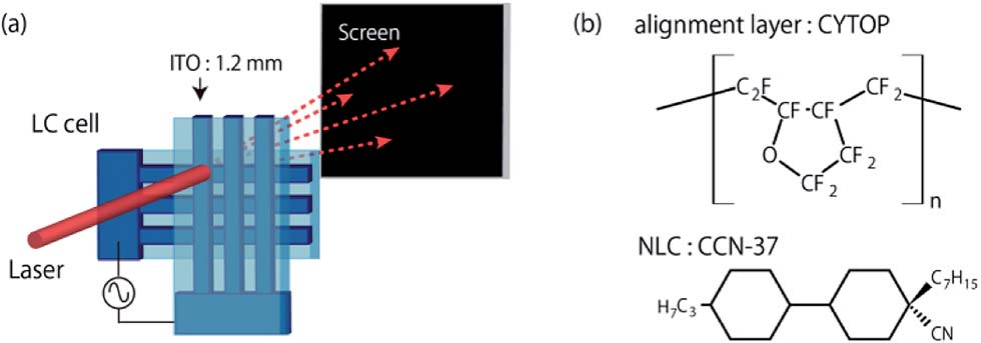
(a) Schematic illustration of the sample cell and the diffraction experiments. (b) The chemical structures of the alignment layer (CYTOP) and the NLC (CCN-37).

Fig. 1 shows the schematic illustration of a sample cell. Using two ITO (indium tin oxide)-coated glass plates, standard sandwich type cells are prepared. The ITO coating is stripe patterned with 1.2 mm wide and the substrates are spin-coated with a perfluoropolymer (CYTOP, Asahi Glass Co.). The thickness of the alignment layer is around 120 nm. Two substrates are overlapped so that the ITO stripes cross orthogonally each other. The mono-dispersed micro-spheres are used to maintain a proper thickness of the sample. From our earlier work, it is known that the obtained grid size is proportional to the sample thickness.^30^ Here, we use the sample cells with the gap of 13 μm and 21 μm. An NLC (4α,4′α-propylheptyl-1α,1′α-bicyclohexyl-4β-carbonitrile, CCN-37, Fig. 1(b)) doped with 1 wt% of an ionic additive (tetrabutylammonium benzoate, TBABE) is filled into the cell by capillary action. CCN-37 possesses a negative dielectric anisotropy (Δ*ε* ∼ −7) and the birefringence (Δ*n*) is 0.03.^36^ At room temperature, CCN-37 shows the homeotropic alignment at the CYTOP surface.^37^ Then, by applying an ac electrical voltage *V* = *V*_0_ cos(2π*ft*), the director reorientation leads to the formation of a square array of umbilical defects in our system. A monodomain can be obtained by adjusting the electrical voltage. We use a He–Ne laser (633 nm in wavelength) to investigate the diffraction property. The monodomain formed in the area of 1.2 mm × 1.2 mm is large enough compared to the beam size of 0.7 mm in diameter. If necessary, wave plates and/or polarizers are placed in front of and behind the sample cell. The power of the diffracted light is measured with a power meter (Thorlabs PM100 USB) for each spot which is seen on the screen. The diffraction is captured with a CMOS camera (Thorlabs DCC1240C). Before starting the diffraction measurements, both the texture of the pattern and the irradiation position of laser beam are checked with polarized optical microscopy (POM). The microscopy is installed on the optical path so that the POM observation is possible without disarranging the optical setup.

## Results and discussion

### Experimental results

**Fig. 2:**
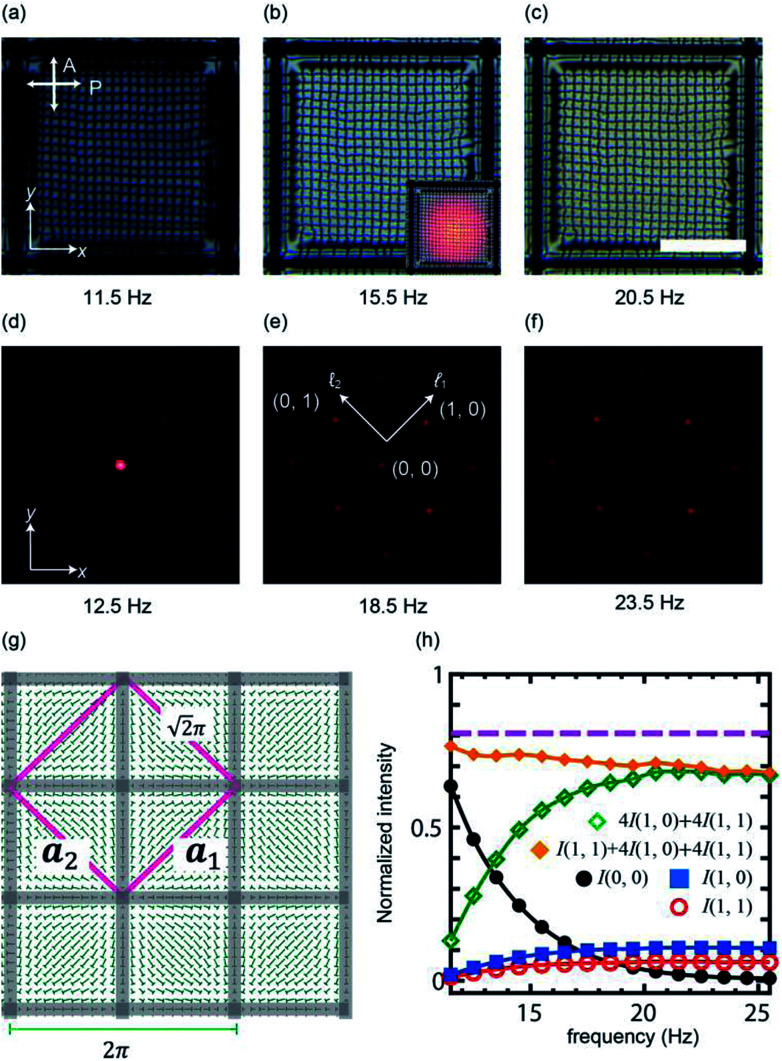
(a)–(c) The micrographic images for the grid-like texture under cross-polarized condition. Scale bar, 500 μm. The inset of (b) is a micrograph of a grid-like texture irradiated with a laser beam. (d)–(f) Diffraction patterns obtained by a laser beam irradiation. The incident beam is circularly polarized. The amplitude of the applied voltage is 25 V. (d) 12.5 Hz, (e) 18.5 Hz, (f) 23.5 Hz. (g) A schematic illustration of the director field of the grid-like texture. The square is the unit cell for the light diffraction. Here, we set the lattice constant as 
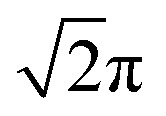
 without loss of generality. (h) The intensity for each spot as a function of frequency. The intensity is normalized with the incident beam. The dashed line is the total power of the light transmitted through the cell. The power meter is placed right behind the cell.

A POM image of the pattern is displayed in Fig. 2(a). The cell thickness is 13 μm. To induce the director reorientation, we increase the frequency, *f* under a constant amplitude, *V*_0_. The range of *f* is chosen not to break the pattern. The change in the micrographic appearance by the increase of *f* is shown in Fig. 2(b) and (c). Due to the enhanced retardation, the brightness changes under the same illumination condition. After preparing the suitable monodomain, a laser beam is irradiated to the sample cell. The inset of Fig. 2(b) is an image at which a linearly polarized laser beam is irradiated. The position of the laser beam on the pattern is checked. We see that the texture of the irradiated area is not damaged, indicating that the heating effect is negligible. The diffraction pattern obtained by a circularly polarized light is shown in Fig. 2(d)–(f). We can see a square array of spots whose direction is tilted by 45° from the horizontal axis. This clearly indicates that the primitive cell of the diffraction pattern corresponds to the square region displayed in Fig. 2(g) as already suggested in our recent study.^35^ We denote the primitive cell vectors by ***a***_1_, ***a***_2_. The primitive reciprocal vectors ***b***_1_ and ***b***_2_ are defined as ***a***_*i*_·***b***_*j*_ = 2π*δ*_*ij*_: ***a***_*i*_ is parallel to ***b***_*i*_ in our case. With ***b***_1_ and ***b***_2_, the spot (*ℓ*_1_, *ℓ*_2_) is expressed as *ℓ*_1_***b***_1_ + *ℓ*_2_***b***_2_ as shown in Fig. 2(e). Here we focus on nine spots {(0, 0), (±1, 0), (0, ±1), (±1, ±1), (±1, ∓1)}. We do not study higher order spots because their intensity is quite weak. With a constant *V*_0_, the effect of the frequency for the diffraction pattern is shown in Fig. 2(d)–(f). It is seen that the intensity at each spot exhibits the frequency dependence. In the low frequency, *i.e.*, in the beginning of the pattern formation from the homeotropic alignment, the zeroth-order spot *I*(0, 0) is dominant. This is obvious because the director field is almost perpendicular to the cell substrate. As the frequency increases, the intensity *I*(0, 0) decreases, while surrounding spots become reinforced. For ∼20 Hz, the zeroth-order diffraction spot almost disappears (Fig. 2(f)), *i.e.*, a high diffraction efficiency is realized. Further increase of *f* does not change the brightness of the spots. This indicates that the director in the cell is well oriented and the retardation cannot be increased further in this cell. To obtain the quantitative data, the intensity of the spots is plotted as a function of frequency. (Fig. 2(h)) The intensity is normalized with that of the incident light (40 μW). As seen from the diffraction pattern, *I*(0, 0) decreases. For higher frequency (≥20 Hz), the change in the intensity is small. A crude estimation is made for how much light is split. We approximate the total power of the diffracted light as *I*(0, 0) + 4(*I*(1, 0) + *I*(1, 1)). The behavior of 4(*I*(1, 0) + *I*(1, 1)) which is the surrounding bright spots, demonstrates that *I*(0, 0) is diffracted to the surrounding spots efficiently. Considering that the transmitted light behind the substrate amounts around 80%, we find that 87% of the transmitted light is diffracted for the surrounding eight spots when *I*(0, 0) takes the minimum value and the rest amount is used for higher-order spots.

**Fig. 3:**
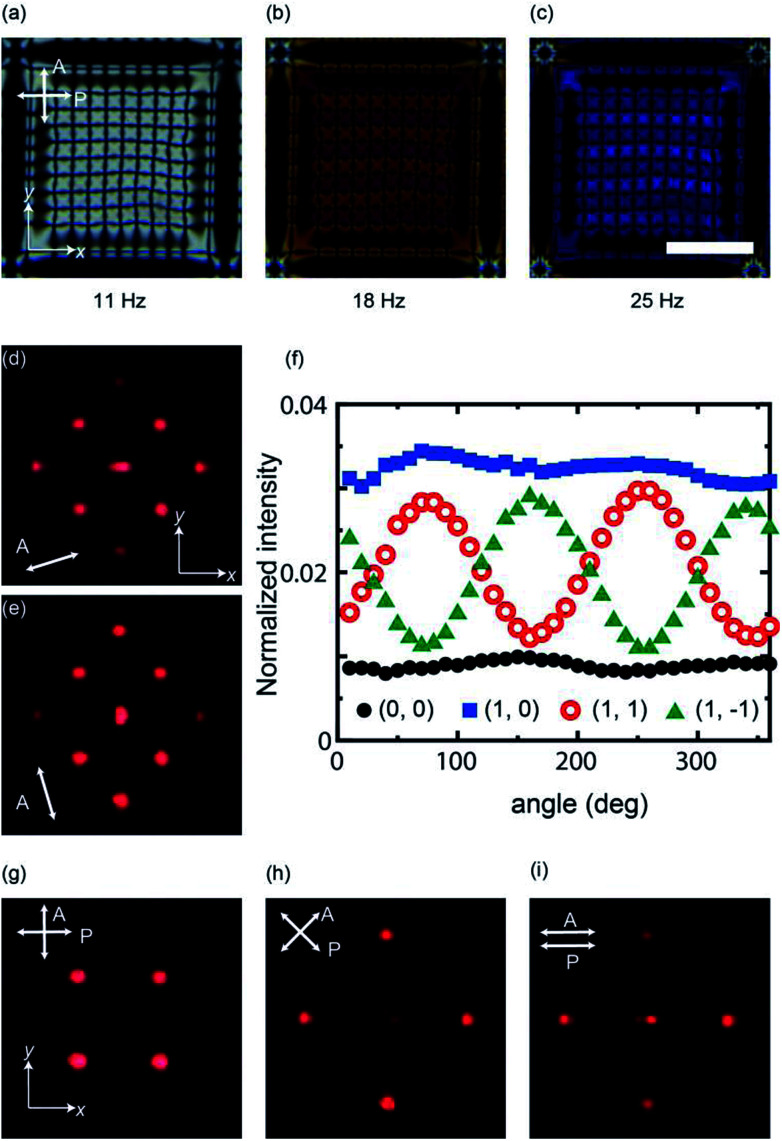
(a)–(c) POM images for a cell with the sample thickness of 21 μm. *V*_0_ = 25 V. *f* = 11 Hz for (a), 18 Hz for (b), and 25 Hz for (c). Scale bar, 500 μm. (d) and (e) Diffraction patterns obtained by rotating the analyzer. The incident light is circularly polarized. (f) The angle dependence for the intensity of diffraction spots. Note that the experimental conditions in (f) and the other diffraction patterns ((d) and (e)) are different. (g)–(i) Diffraction patterns obtained by using a linearly polarized light.

In addition to the effect of the electrical voltage for the light intensity, the polarization properties are studied by placing an analyzer behind the sample cell. Typical results are summarized in Fig. 3, which uses a cell with the thickness of 21 μm. POM observation under crossed-polarizers shows that the size of the grid becomes large compared to Fig. 2 because it is proportional to the sample thickness.^30^ By increasing the frequency, POM images show a significant color alternation due to the change of the retardation. (Fig. 3(b) and (c)) Considering the birefringence of Δ*n* ∼ 0.03, the possible maximum value of Δ*nd* is ∼600 nm if the director configuration is planar. This can be seen as a blue color under POM, which qualitatively agrees with the observation. This suggests that the director is substantially tilted in the sample cell around 25 Hz. The incident light is circularly polarized as used in Fig. 2. The diffraction pattern exhibits a similar trend to that of 13 μm cell. When the director tilts from the homeotropic alignment, the value of *I*(0, 0) approaches zero because the light is split. (ESI Fig S1†) An additional feature observed for the 21 μm-thick cell is that the decreased *I*(0, 0) is reinforced from the minimum value by increasing the frequency. This is because the retardation can be varied over a wide range compared to the 13 μm-thick cell. Fig. 3(d) and (e) show that the brightness changes depending on the direction of analyzer. We notice that there are two types of spots whose intensity is independent {(0, 0), (±1, 0), (0, ±1)} and dependent {(±1, ±1), (±1, ∓1)} from the direction of analyzer. This means that the polarization of the diffracted light is converted differently depending on the spots. We also see that the quality of the diffracted spots is slightly low compared to the 13 μm-thick cell. For example, the zeroth order spot is distorted with the direction of the analyzer. However, this is due to the imperfection of the grid-like structure and the decreased number of the grids in the irradiated area. In order to investigate details, the frequency is adjusted at a moderate value so that all the spots exhibit comparable brightness. The intensity at each spot is plotted as a function of the direction of analyzer in Fig. 3(f). We note that the low value in the normalized intensity is due to the presence of the polarizers which absorb the light. For the spots of {(0, 0), (±1, 0), (0, ±1)}, we can expect that the diffracted light is circularly polarized. On the other hand, the spots of {(±1, ±1), (±1, ∓1)} indicate that the polarization is elliptical. For these four spots, the angular dependence of the intensity of (±1, ±1) and (±1, ∓1) is opposite, *i.e.*, if *I*(±1, ±1) decrease, *I*(±1, ∓1) increase. The phase shift for {(±1, ±1), (±1, ∓1)} varies by the applied electric field. Further experiments are carried out using a linearly polarized light. The diffraction pattern is studied under cross- and parallel-polarized conditions. (Fig. 3(g)–(i)) The zeroth-order spot can be erased as far as the cross-polarized condition is kept. For the other diffraction spots, the brightness changes by rotating crossed-polarizers. If a polarizer is set parallel to *x*-axis, four spots at (±1, 0), (0, ±1) are obtained. On the other hand, when the crossed-polarizers are rotated by 45°, the spots (±1, 0), (0, ±1) disappear and the spots of (±1, ±1), (±1, ∓1) appear. Thus, *I*(0, 0) and other spots (±1, 0), (0, ±1) which are independent of the direction of analyzer (Fig. 3(f)), have different property of polarization conversion. The different conversion can be found under parallel polarized condition. We find that *I*(±1, 0) and *I*(0, ±1) cannot be observed. These observations indicate that the conversion at (±1, 0) and (0, ±1) is the same as that of the half-wave plate whose optical axis is directed to the origin.

### Numerical calculation

To understand the observation, we calculate the diffraction numerically by using the Jones matrix method. This approach is valid because the diffraction angle of this experiment is typically around 0.01 rad. Firstly, we estimate the effective retardation in two dimension and map it to the projected director field. It is to be noted that the maximum retardation which is the value at the center of each grid, increases together with the spatial change of the director field by increasing the frequency. Then, to describe the frequency dependence, we express the retardation as Δ*nd* = (*n*_e_^eff^(***r***) − *n*_0_)*d* = *δ*_0_*F*(***r***) = *δ*(***r***) where ***r*** = (*x*, *y*). *δ*_0_ is the maximum value of the retardation at the center of each grid and *F*(*x*, *y*) is a function which qualitatively reproduces the effect of the director tilts. Supposing that the cell thickness is 20 μm, *δ*_0_ ≤ 600 nm and 0 ≤ *F*(***r***) ≤ 1 are imposed. Using Jones matrix *J*(***r***) for the LC cell, the diffraction pattern at the screen is calculated with ***E*~**(***k***) ∝ ∫*J*(***r***)***E***(***r***)exp(−*i****k***·***r***)*d****r***. We assume that the incoming laser beam ***E***(***r***) is uniform over the irradiated area, *i.e.*, ***E***(***r***) = ***E***_in_. Then ***E*~**(***k***) ∝ ∫*J*(***r***)exp(−*i****k***·***r***)*d****r***·***E***_in_. When two optical axes of the director field are parallel to *x*- and *y*-axis, the Jones matrix *J*′ is given as
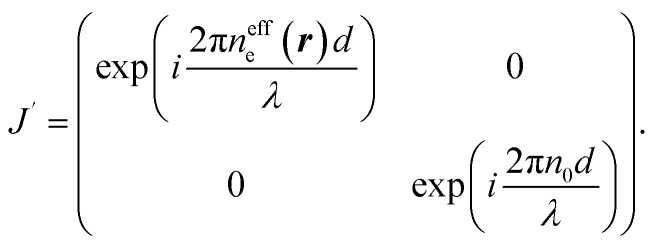
*n*_e_^eff^(***r***) varies depending on ***r***. Then we obtain1
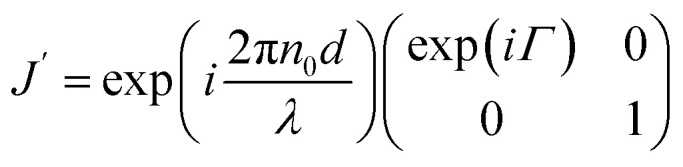
where *Γ* = 2πΔ*nd*/*λ*. By neglecting the constant term, *J*(***r***) is expressed as2
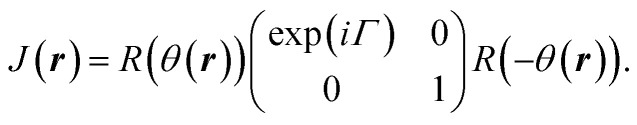


Here *θ*(***r***) is the rotation angle of the director from *x*-axis. *R*(*θ*) is the rotation matrix, which is given as
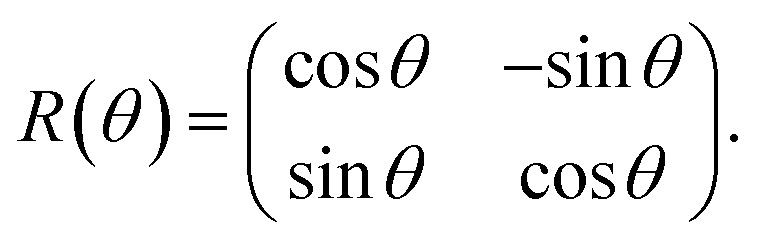


Using (*n*_*x*_, *n*_*y*_), we obtain tan^−1^(*n*_*y*_/*n*_*x*_). To describe the director field shown in Fig. 2(g), we use a vector field of(*n*_*x*_, *n*_*y*_) ∝ (sin *x*, sin *y*).

**Fig. 4:**
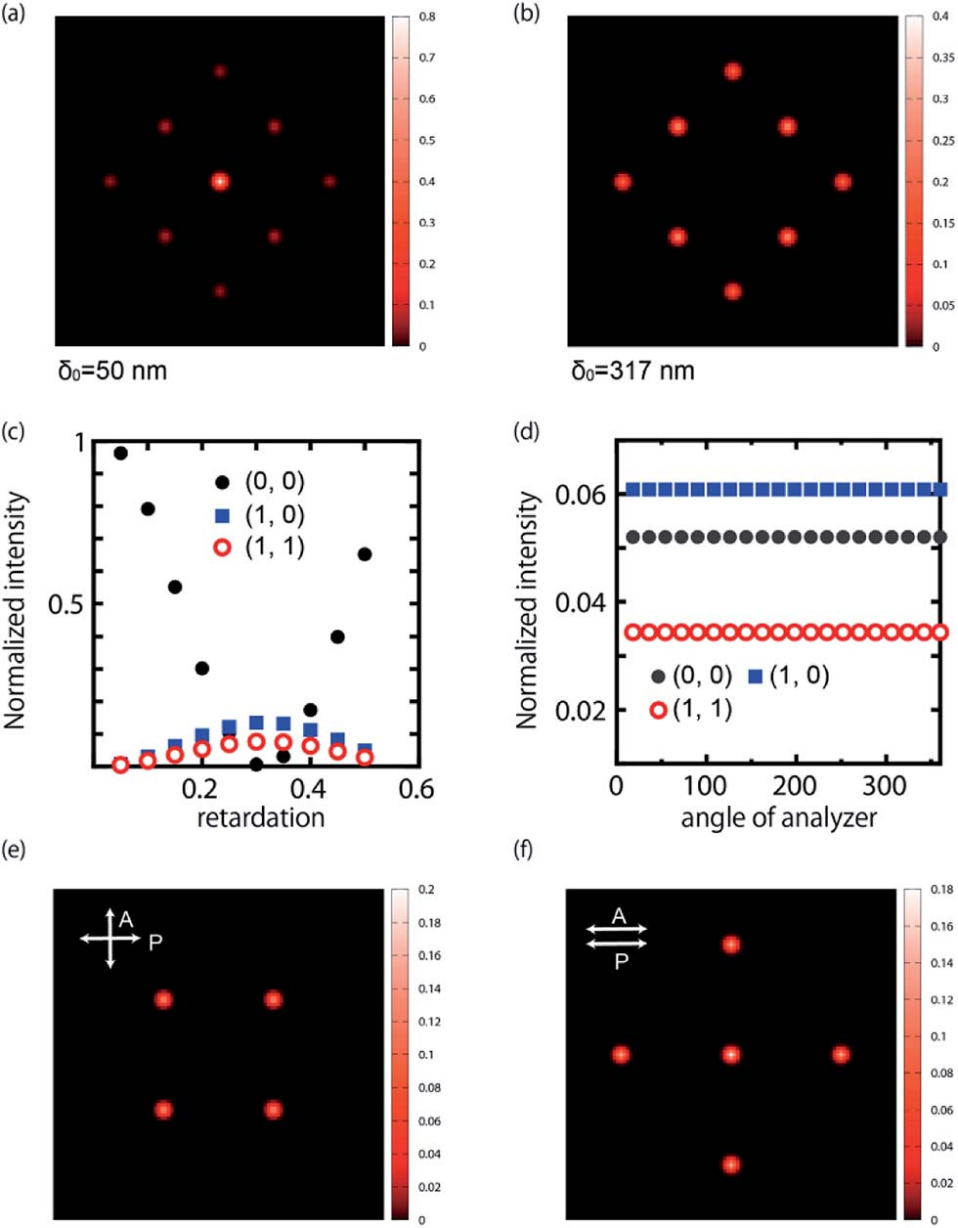
Calculated diffraction patterns for the planar alignment condition using circularly polarized input light and retardation of (a) 50 and (b) 300 nm. (c) The behavior of the intensity depending on the retardation. (d) The polarization dependence for the spot. Here the calculation is made for Δ*nd* = 250 nm. (e) and (f) Calculated diffraction patterns under crosspolarized (e) and parallel polarized (f) conditions.

First, we examine the case for the constant retardation of *F*(*x*, *y*) = 1, *i.e.*, Δ*nd* = *δ*_0_. This basically corresponds to the planar alignment with defects. Some calculated diffraction patterns are plotted in Fig. 4(a) and (b), where circularly polarized input light is used. The appearance seems to reproduce the experiments. We change the value of the retardation, which qualitatively corresponds to the application of the electric field. (Fig. 4(c)) We find that the diffraction efficiency changes depending on the retardation of the sample. For small *δ*_0_, the zeroth-order spot *I*(0, 0) is dominant and the surrounding spots are weak. At 2*δ*_0_ = *λ*, *I*(0, 0) vanishes and the diffraction efficiency reaches 100%. The surrounding spots increase the intensity. When *δ*_0_ is increased further, *I*(0, 0) increases again and the surrounding spots become darker. The intensity for (1, 0) is higher than that for (1, 1). The tendency agrees with the experiments. On the other hand, a contrast is found in the polarization properties (Fig. 4(d)). The intensity at each spot is obtained by placing analyser behind the sample. In contrast to the experiments, the calculation does not show the angular dependence of the rotating analyser for all the spots. This means that the split light is circularly polarized even for (1, ±1) and (−1, ±1) when the alignment is planar. The calculated diffraction pattern under crossed- and parallel-polarizers is shown in Fig. 4(e) and (f). The appearance is similar to that of the experiment. Thus, we notice that the spots of (±1, 0) and (0, ±1) behave as a half wave plate whose optical axis is directed to *x*- and *y*-axes. Thus, as suggested from the experiments, when the polarization of the incident light is parallel to *x*-axis, it can pass through the analyzer even in a cross-polarized condition. From the above, we find that the planar alignment condition qualitatively explains the experiments, while the obtained numerical results are incomplete in part.

**Fig. 5:**
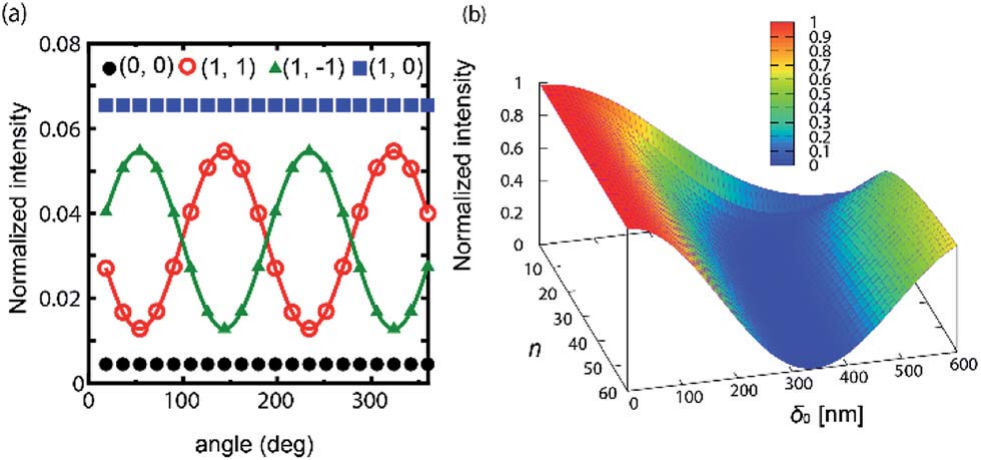
(a) The angular dependence of the intensity of diffraction spots. The value of intensity is normalized with that of the incident light. The calculation is made with *δ*_0_ = 350 nm and *n* = 40. (b) The intensity of *I*(0, 0) as a function of *n* and *δ*_0_.

In our experimental system, the retardation varies depending on the reorientation of the director. To evaluate the influence of umbilical defects qualitatively, we use a function *F*(*x*, *y*) = (2 −cos^*n*^ *x* − cos^*n*^ *y*)/2 for effective value. *n* takes an even number. For a small *n*, this corresponds to the beginning of the formation of the grid-like texture, while further tilted configuration can be described with a larger *n*. We consider the effects by changing *δ*_0_ and *n*. In Fig. 5(a), we show a calculation which is made with *δ*_0_ = 350 nm and *n* = 40. An important finding is that for (±1, ±1), (±1, ∓1), the polarization property is modified, which agrees with the experiments. This means that the elliptically polarized light at these spots is due to the spatial modulation of the birefringence. Moreover, from the simulated results, we can elucidate the director field of the sample. For the presented case, it is suggested that the director field changes suddenly near the core of the defect and the other region has a uniform tilt. We also check the behavior of *I*(0, 0), which is plotted as the function of *δ*_0_ and *n*. (Fig. 5(b)) When *n* has a larger value, the graph shows a minimum. In particular, *I*(0, 0) becomes close to 0 for a high value of *n*. Similar to the case of the planar configuration (Fig. 4(c)), we notice that *I*(0, 0) exhibits a minimum point around *δ*_0_ ∼ *λ*/2. This demonstrates that our experimental system can also realize nearly 100% diffraction efficiency.

### Theoretical consideration

**Fig. 6:**
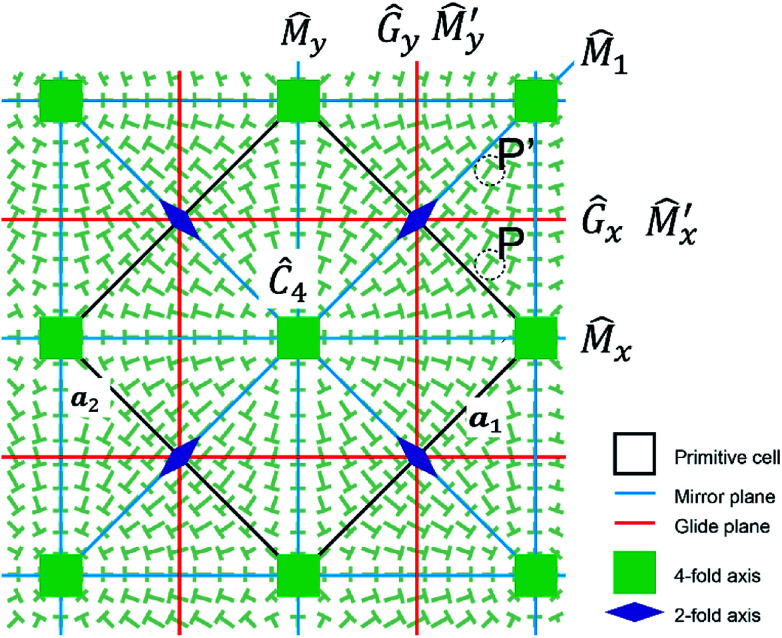
Schematic illustration of the symmetry operation for this system.

We discuss the results of diffraction patterns from a symmetrical point of view. Taking into account that two director states (*n*_*x*_, *n*_*y*_, *n*_*z*_) and (*n*_*x*_, *n*_*y*_, −*n*_*z*_) are optically the same, *i.e.*, the corresponding Jones matrices are the same, the grid-like pattern belongs to the two-dimensional space group *P*4*m*. The symmetry elements are shown in Fig. 6. The symmetry operation can be expressed as ***r***′ = *T̂****r*** = *T****r*** + ***t***, where *T* is a matrix representing a rotation or a mirror (reflection) with respect to the origin, and ***t*** is a translation vector. A Jones matrix *J*(***r***) is transformed to *TJ*(***r***)*T*^−1^ by *T̂* as the Jones matrices are second rank tensors, and ***r*** is moved to *T****r*** + ***t***. Since *T̂* is a symmetry operator, we have

3 *J*(*T****r*** + ***t***) = *TJ*(***r***)*T*^−1^

The Jones matrix in the Fourier space is defined as4
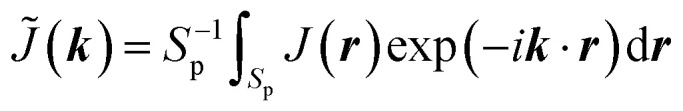
where the integration is taken over the primitive cell defined by two primitive vectors ***a***_1_ and ***a***_2_ shown in Fig. 6 and *S*_p_ is its area. Fourier transformation of eqn (3) yields

5 *J̃*(*T****k***)exp(−*iT****k***·***t***) = *TJ̃*(***k***)*T*^−1^

In the following, we examine the symmetry of *J̃*(***k***) at some ***k*** based on eqn.^5^ For simplicity, we use the director field of Fig. 2(g) because the following results are valid independent of specific director fields as far as the symmetry is *P*4*m*. First, we consider a symmetry operation with a mirror *M̂*_1_ (*i.e.*, *T̂****r*** = *M̂*_1_***r*** = *M*_1_***r*** + ***t***) which is the diagonal direction in Fig. 6. Here, *M*_1_ and ***t*** are given as
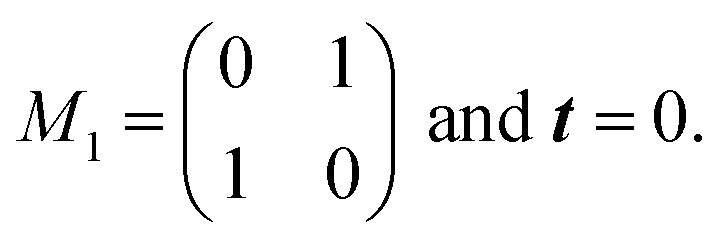


For ***k*** = ***b***_1_, we notice that *M*_1_***b***_1_ = ***b***_1_. By using
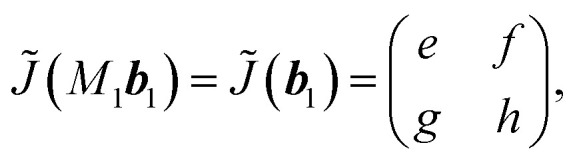
eqn (5) becomes
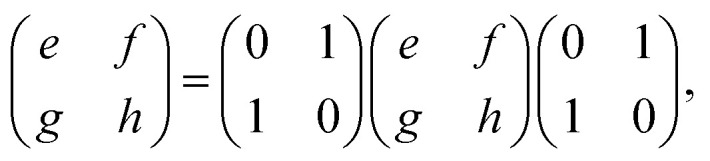
which leads to6
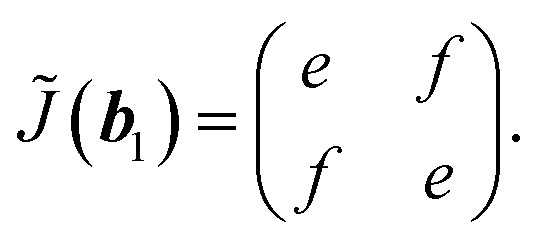


The symmetric form of Jones matrix indicates that for the diffracted light at ***b***_1_ the sample plays a role of a wave-plate whose principal axes (the optical axes) are diagonal between *x*- and *y*-axes. To obtain *J̃*(−***b***_2_), we use another symmetry operation which rotates the director by 
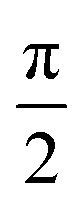
 with respect to *z*-axis. (*Ĉ*_4_) Taking ***k*** = ***b***_1_, we obtain from eqn (5)7
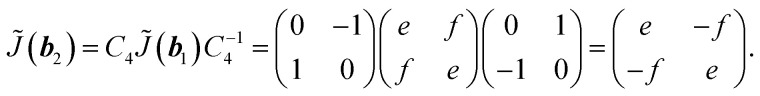


In the same way, the operation of *Ĉ*_4_ can be applied for *J̃*(−***b***_1_) and *J̃*(−***b***_2_), which leads to

8 *J̃*(−***b***_1_) = *J̃*(***b***_1_)

9 *J̃*(−***b***_2_) = *J̃*(***b***_2_)

Second, we consider ***k*** = ***b***_1_ − ***b***_2_. Using a mirror operator in *x*-direction (*M̃*_*x*_), eqn (5) gives10
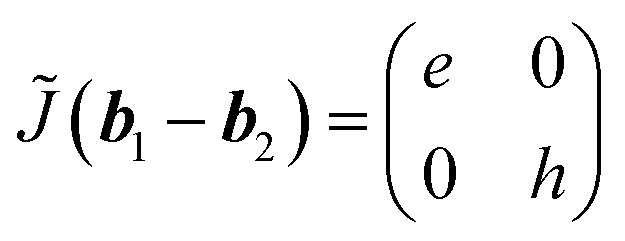
and further successive operation of *Ĉ*_4_ gives rise to the followings:11
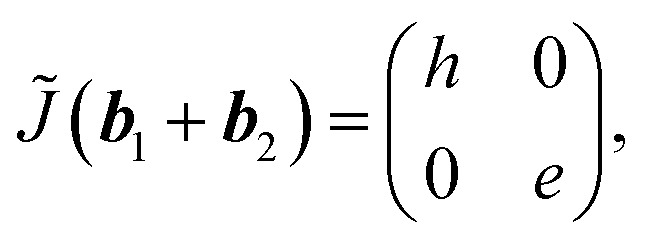

12 *J̃*(−***b***_1_ + ***b***_2_) = *J̃*(***b***_1_ − ***b***_2_),

13 *J̃*(−***b***_1_ − ***b***_2_) = *J̃*(***b***_1_ + ***b***_2_).

At these diffraction spots, the optical axes are in *x*- and *y*-directions.

Last, we show only the result at ***k*** = 0,
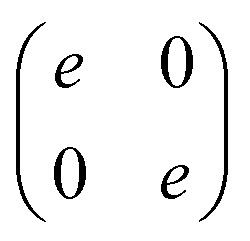
which gives optically isotropic properties.

From experiments and numerical calculations, for ***k*** = ***b***_1_, the property of the polarization conversion is the same as that of the half-wave plate whose optical axes are directed to the origin. This indicates that *e* of *J̃*(***b***_1_) in eqn (6) should vanish. The above-mentioned symmetry never leads to the expected result. However, we notice another symmetry (this is not usual symmetry) in Fig. 6, that is, the tilt angles at *P* and *P*′ are the same, which is mutually exchanged by the mirror 
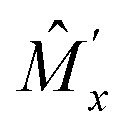
 (note that 
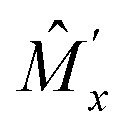
 is different from the glide *Ĝ*_*x*_, although they are in the same line.). 
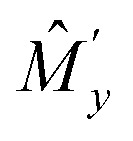
 is also this type of operator. In general, these operators do not exist although they do in our case of (*n*_*x*_, *n*_*y*_) ∝ (sin *x*, sin *y*). It is easily confirmed that they exist for (*n*_*x*_, *n*_*y*_) ∝ (*g*(*x*), *g*(*y*)) with 
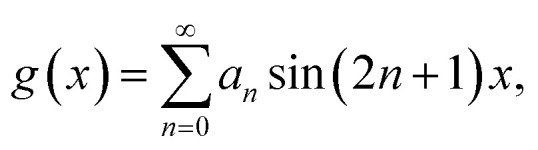
 where *a*_*n*_ are arbitrary constants. For 
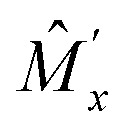
 the following equation is valid instead of eqn (5) as *J̃* does not change by 
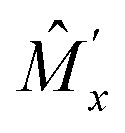
14
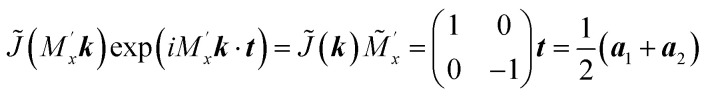


Substitution of ***k*** = ***b***_1_ into eqn (14) yields −*J̃*(−***b***_2_) = *J̃*(***b***_1_). Using eqn (6), (7) and (9), we finally obtain *e* = 0, that is, the Jones matrix of the half-wave plate:15
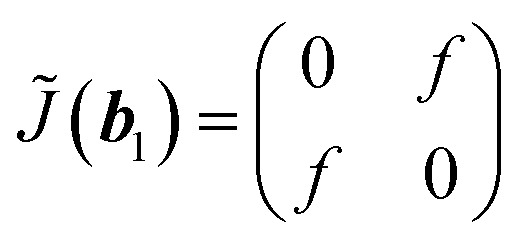


The symmetry also allows us to reduce *J̃*(***k***), when we calculate the integration in eqn (4) by using eqn (2). We assume that *θ*(***r***) and *δ*(***r***) have the symmetry of *P*4*m*. The non-vanishing elements are given as
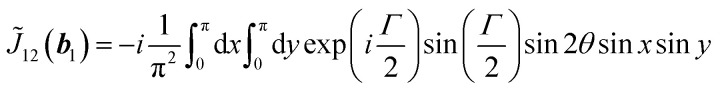

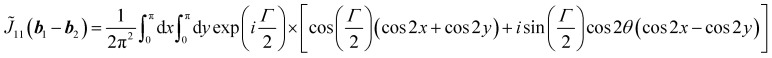

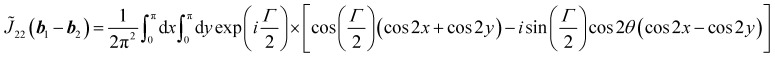

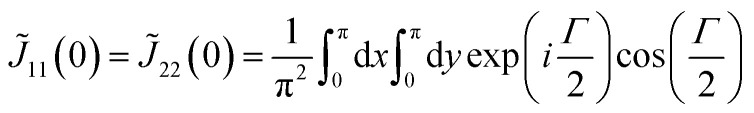
when *δ* is constant and tan *θ* = sin *y*/sin *x*, corresponding to high voltage states, the above equations are reduced;16
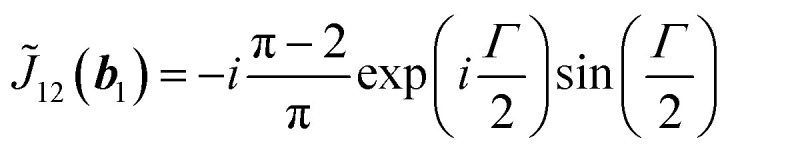
17
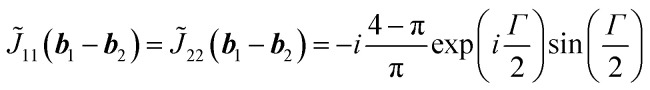
18
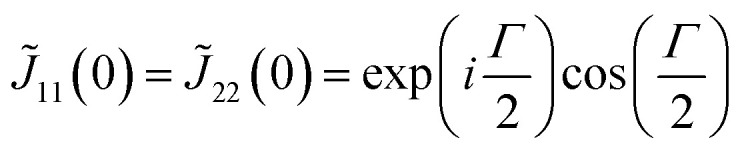


Particularly, from eqn (17), it is seen that the diffraction at ***k*** = ***b***_1_ − ***b***_2_ plays a role of the half-wave plate as well as that at ***k*** = ***b***_1_. In other words, a feature of the spot at ***b***_1_ − ***b***_2_ is that the elliptical polarization can be tuned by the electrical voltage. However, since the grid-like structure simultaneously becomes unstable under a high-voltage, the realization of the tunability is a future work.

## Conclusions

To conclude, we have reported how light is diffracted on a two-dimensional micro-structure of nematic liquid crystals. Because the structure is obtained by self-organization, the pattern works as an electrically tunable as well as switchable optical grating. Under a moderate electrical voltage, the intensity of the zeroth-order diffraction spot can be tuned, which realizes a high diffraction efficiency. In addition, it is demonstrated that the polarization of the incident light can be converted to different kinds depending on the location of spots. The numerical and theoretical calculations also reproduce well the experiments. Particularly, at some spots, this pattern has a function that inverts the handedness of the circularly polarized light. From the theoretical analysis, it is found that the symmetry of the director field plays important roles for the type of polarization of the diffracted light. These results suggest that further investigations can be performed by changing the birefringence of NLCs and by changing the pattern. Moreover, the combination with the polymer-stabilization technique is interesting. This allows us not only to maintain the director field but also tune the optical properties by external stimuli such as temperature and electric fields,^4,7^ which can be applied in the present system.^38^

## Conflicts of interest

There are no conflicts to declare.

## Supplementary Material

RA-008-C8RA08557A-s001

## References

[cit1] Chen J., Bos P. J., Vithana H., Johnson D. L. (1995). Appl. Phys. Lett..

[cit2] Subacius D., Bos P. J., Lavrentovich O. D. (1997). Appl. Phys. Lett..

[cit3] Kawatsuki N., Hasegawa T., Ono H., Tamoto T. (2003). Adv. Mater..

[cit4] Ren H., Fan Y.-H., Wu S.-T. (2003). Appl. Phys. Lett..

[cit5] Ono H., Emoto A., Takahashi F., Kawatsuki N., Hasegawa T. (2003). J. Appl. Phys..

[cit6] Senyuk B. I., Smalyukh I. I., Lavrentovich O. D. (2005). Opt. Lett..

[cit7] Yan J., Li Y., Wu S.-T. (2011). Opt. Lett..

[cit8] Chen H., Tan G., Huang Y., Weng Y., Choi T.-H., Yoon T.-H., Wu S.-T. (2017). Sci. Rep..

[cit9] Park J.-H., Yu C.-J., Kim J., Chung S.-Y., Lee S.-D. (2003). Appl. Phys. Lett..

[cit10] Le Doucen M., Pellat-Finet P. (1998). Opt. Commun..

[cit11] Xu D., Tan G., Wu S.-T. (2015). Opt. Express.

[cit12] Ryabchun A., Bobrovsky A., Gritsai Y., Sakhno O., Shibaev V., Stumpe J. (2015). ACS Appl. Mater. Interfaces.

[cit13] He Z., Nose T., Sato S. (1996). Jpn. J. Appl. Phys., Part 1.

[cit14] Zhu J.-L., Lu J.-G., Qiang J., Zhong E.-W., Ye Z.-C., He Z., Guo X., Dong C.-Y., Su Y., Shieh H.-P. D. (2012). J. Appl. Phys..

[cit15] Wen B., Petschek R. G., Rosenblatt C. (2002). Appl. Opt..

[cit16] Gibbons W. M., Sun S. (1994). Appl. Phys. Lett..

[cit17] Yoshida H., Asakura K., Fukuda J., Ozaki M. (2015). Nat. Commun..

[cit18] Guo Y., Jiang M., Peng C., Sun K., Yaroshchuk O., Lavren-tovich O., Wei Q. H. (2016). Adv. Mater..

[cit19] Jau H.-C., Lin T.-H., Chen Y.-Y., Chen C.-W., Liu J.-H., Fuh A. Y.-G. (2012). Appl. Phys. Lett..

[cit20] Xiang Y., Jing H.-Z., Zhang Z.-D., Ye W.-J., Xu M.-Y., Wang E., Salamon P., Éber N., Buka Á. (2017). Phys. Rev. Appl..

[cit21] Vaupotič N., Ali M., Majewski P. W., Gorecka E., Pociecha D. (2018). ChemPhysChem.

[cit22] Meyer R. B. (1969). Phys. Rev. Lett..

[cit23] Pieranski P., Dubois-Violette E., Guyon E. (1973). Phys. Rev. Lett..

[cit24] Kuzma M. R. (1986). Phys. Rev. Lett..

[cit25] Buka A. and Kramer L., Pattern formation in liquid crystals, Springer New York, New York, NY, 1996

[cit26] Migara L. K., Song J.-K. (2018). NPG Asia Mater..

[cit27] Yeh H.-C., Chen G.-H., Lee C.-R., Mo T.-S. (2007). J. Chem. Phys..

[cit28] Kang S.-W., Chien L.-C. (2007). Appl. Phys. Lett..

[cit29] Ryabchun A., Bobrovsky A. (2018). Adv. Opt. Mater..

[cit30] Sasaki Y., Jampani V., Tanaka C., Sakurai N., Sakane S., Le K. V., Araoka F., Orihara H. (2016). Nat. Commun..

[cit31] Rapini A. (1973). J. Phys..

[cit32] Rapini A., Léger L., Martinet A. (1975). J. Phys. Colloq..

[cit33] Brasselet E., Loussert C. (2011). Opt. Lett..

[cit34] Brasselet E. (2012). Phys. Rev. Lett..

[cit35] Salamon P., Éber N., Sasaki Y., Orihara H., Buka Á., Araoka F. (2018). Phys. Rev. Appl..

[cit36] Oswald P., Poy G., Dequidt A. (2017). Liq. Cryst..

[cit37] Dhara S., Kim J. K., Jeong S. M., Kogo R., Araoka F., Ishikawa K., Takezoe H. (2009). Phys. Rev. E: Stat., Nonlinear, Soft Matter Phys..

[cit38] Sasaki Y., Ueda M., Le K. V., Amano R., Sakane S., Fujii S., Araoka F., Orihara H. (2017). Adv. Mater..

