# Supplementary material for: Tunable two-dimensional polarization grating using a self-organized micropixelated liquid crystal structure
Source: RSC Adv. 2018 Dec 12;8(72):41472–9. doi: 10.1039/c8ra08557a (PMC9092010; doi:10.1039/c8ra08557a)
Supplement: RA-008-C8RA08557A-s001 [file RA-008-C8RA08557A-s001.pdf]

Electronic Supplementary Material (ESI) for RSC Advances.
This journal is © The Royal Society of Chemistry 2018

Electronic Supplementary Information (ESI)

Reo Amano,[1] Péter Salamon,[2] Shunsuke Yokokawa,[1] Fumiaki Kobayashi,[1] Yuji Sasaki,[1*] Shuji Fujii,[1] Ágnes Buka,[2] Fumito Araoka,[3] and Hiroshi Orihara[1]

1 Division of Applied Physics, Faculty of Engineering, Hokkaido University North 13 West 8, Kita-ku, Sapporo, Hokkaido 060-8628 (Japan)

2 Institute for Solid State Physics and Optics, Wigner Research Centre for Physics, Hungarian Academy of Sciences, H-1525 Budapest, P.O.B.49 (Hungary.)

3 RIKEN Center for Emergent Matter Science (CEMS), 2-1 Hirosawa, Wako, Saitama 351-0198 (Japan)

[*] Corresponding author: yuji.sasaki@eng.hokudai.ac.jp

Figure S1

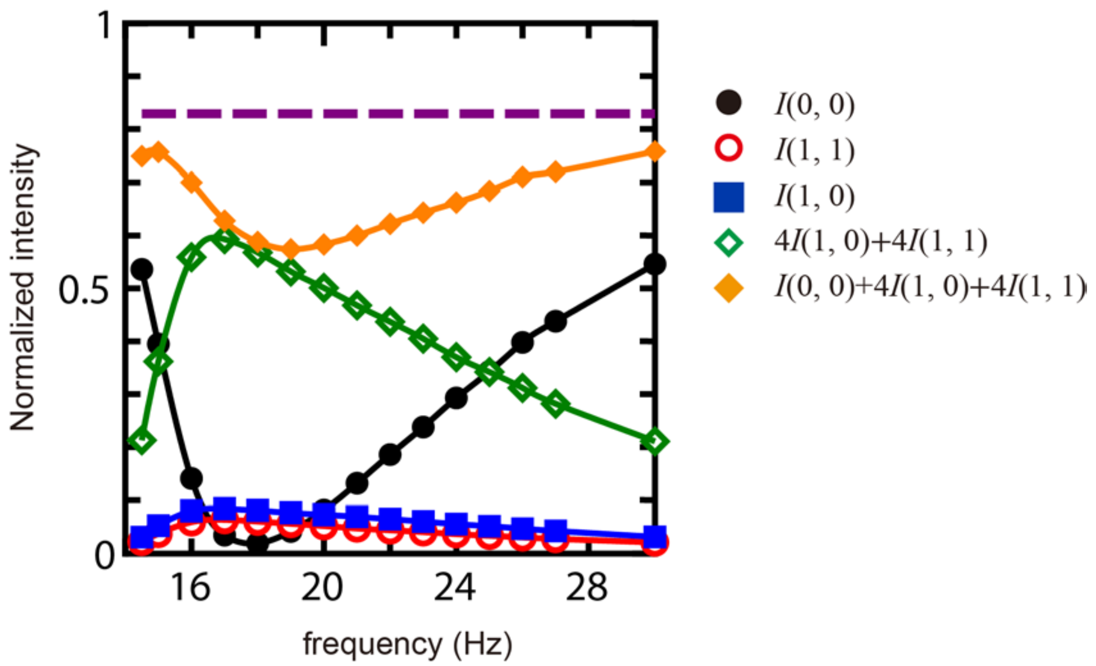

Figure S1. The intensity of diffracted light for each spot as a function of frequency. The sample thickness is 21 μm. The intensity is normalized with that of the incident beam. The dashed line is the total power of the light transmitted through the cell. In this case, the power meter is placed right behind the cell.
